# The IRYSS-COPD appropriateness study: objectives, methodology, and description of the prospective cohort

**DOI:** 10.1186/1472-6963-11-322

**Published:** 2011-11-24

**Authors:** José M Quintana, Cristóbal Esteban, Irantzu Barrio, Susana Garcia-Gutierrez, Nerea Gonzalez, Inmaculada Arostegui, Iratxe Lafuente, Marisa Bare, Juan Antonio Blasco, Silvia Vidal

**Affiliations:** 1 Unidad de Investigación, Hospital Galdakao-Usansolo - CIBER Epidemiología y Salud Pública (CIBERESP), Galdakao, Bizkaia, Spain; 2 Servicio de Respiratorio, Hospital Galdakao-Usansolo, Galdakao, Spain; 3 Departamento de Matemática Aplicada, Estadística e Investigación Operativa- CIBER Epidemiología y Salud Pública (CIBERESP), Universidad del País Vasco, Lejona, Bizkaia, Spain; 4 Unidad de Epidemiología Clínica, Corporacio Parc Tauli, Barcelona, Spain; 5 Agencia Lain Entralgo, Madrid, Spain; 6 Unidad de Calidad, Hospital Valme, Sevilla, Spain; 7 Departamento de Medicina Preventiva y Salud Pública, Universidad del País Vasco, Lejona, Bizkaia, Spain

## Abstract

**Background:**

Patients with chronic obstructive pulmonary disease (COPD) often experience exacerbations of the disease that require hospitalization. Current guidelines offer little guidance for identifying patients whose clinical situation is appropriate for admission to the hospital, and properly developed and validated severity scores for COPD exacerbations are lacking. To address these important gaps in clinical care, we created the IRYSS-COPD Appropriateness Study.

**Methods/Design:**

The RAND/UCLA Appropriateness Methodology was used to identify appropriate and inappropriate scenarios for hospital admission for patients experiencing COPD exacerbations. These scenarios were then applied to a prospective cohort of patients attending the emergency departments (ED) of 16 participating hospitals. Information was recorded during the time the patient was evaluated in the ED, at the time a decision was made to admit the patient to the hospital or discharge home, and during follow-up after admission or discharge home. While complete data were generally available at the time of ED admission, data were often missing at the time of decision making. Predefined assumptions were used to impute much of the missing data.

**Discussion:**

The IRYSS-COPD Appropriateness Study will validate the appropriateness criteria developed by the RAND/UCLA Appropriateness Methodology and thus better delineate the requirements for admission or discharge of patients experiencing exacerbations of COPD. The study will also provide a better understanding of the determinants of outcomes of COPD exacerbations, and evaluate the equity and variability in access and outcomes in these patients.

## Background

Chronic obstructive pulmonary disease (COPD) is one of the most common chronic diseases, and its prevalence is expected to increase over the next few decades [1-3]. COPD is a leading cause of death in developed countries, and patients with COPD generally have a substantial deterioration in their quality of life [4,5]. Patients often experience exacerbations of COPD, and these often require hospitalization.

Various guidelines have been developed to provide clinicians with recommendations for managing patients with stable COPD as well as those experiencing exacerbations of the disease [6-8]. However, these guidelines offer little guidance for identifying patients whose clinical situation is appropriate for admission to the hospital. In addition, although some disease severity scores exist for patients with stable COPD, no robust severity score has been developed for patients experiencing an exacerbation [9].

Observational studies have not yet provided conclusive information on steps that can be taken to improve the process of care for patients experiencing exacerbations of COPD, and randomized controlled trials are unlikely to be conducted to shed light on this topic. One method that can be used to address areas of clinical uncertainty was designed by the Research and Development (RAND) Corporation and the University of California-Los Angeles (UCLA). Called the RAND/UCLA Appropriateness Methodology (RAM), it can be used to determine best practices guidelines. It has previously been used to improve clinical decision making in the emergency department (ED) [10].

We used the RAM to create explicit criteria for hospital admission from the ED for patients with COPD exacerbations [11,12]. To validate these criteria in real-world clinical situations, we created the Investigacion en Resultados y Servicios de Salud COPD Appropriateness Study (IRYSS-CAS) group. The goals of this prospective cohort study, which was conducted in 16 hospital EDs, include validating the explicit criteria developed by the RAM, evaluating the variability between hospitals in the appropriateness of hospital admission of patients experiencing COPD exacerbations, and studying variability in access to care and outcomes. We also developed a robust severity scale for patients with COPD exacerbations. In this report, we describe the protocol as well as the methods needed for the collection of data and their management.

## Methods/Design

### Development of appropriateness criteria

We used the RAND/UCLA Apropriateness Methodology[10] to develop criteria for determining the appropriateness of hospital admission for patients experiencing exacerbations of COPD who attended an ED [11,12]. The methodology consists of the following steps:

First, an extensive literature review was conducted to summarize existing knowledge about criteria for hospital admission for patients attending an ED with exacerbations of COPD.

Second, results from this review were used to develop a comprehensive and detailed list of mutually exclusive and clinically specific scenarios regarding hospitalization for patients attending an ED with an exacerbation of COPD. A total of 896 scenarios were created from combinations of the following variables: age, presence of diabetes mellitus, presence of cardiovascular disease, expected adherence to treatment, response to prior treatments, severity of baseline COPD, severity of COPD exacerbation, number of hospital admissions in the year preceding the exacerbation, and need for oxygen therapy at home.

Third, we used a modified Delphi process[11,12] to create appropriateness criteria. We assembled a panel of 7 pneumologists and 5 ED physicians who were nationally recognized in their fields. Candidates' names were provided by their respective medical societies and members of our research team. In the first round of the two-round process, the panelists were mailed the literature review and the 896 scenarios and asked to rate each scenario for the appropriateness of hospital admission, considering the average patient and physician in the year 2007. Panelists were also given the definition of "appropriate" established by the RAM: A procedure or treatment is considered to be appropriate if the expected health benefit exceeds the expected negative consequences by a sufficiently wide margin that the procedure is worth doing, exclusive of cost.

Panelists rated each scenario using a 9-point scale, ranging from 1 point for an extremely inappropriate admission to 9 points for an extremely appropriate admission. Each panelist returned his or her ratings by mail.

Members of the IRYSS-CAS team compiled and collated the ratings. A scenario was defined as appropriate if the panel's median score was between 7 and 9 without disagreement and inappropriate if the median score was between 1 and 3 without disagreement. Scenarios were defined as uncertain if the median score was between 4 and 6 or if the members of the panel disagreed. Disagreement was defined as at least one-third of the panelists rating a scenario from 1 to 3 and at least another third rating it from 7 to 9; agreement was defined as less than one third of the panelists rating an indication outside a 3-point region (1 to 3, 4 to 6, or 7 to 9) containing the median. If neither agreement nor disagreement was found, the scenario was defined as uncertain.

The results of the first round were presented to the 12 panelists at a one-day meeting. During that meeting, the panelists discussed variables and points of disagreement, and rated the scenarios again. As before, members of the IRYSS-CAS team compiled and collated the ratings and created the final list of appropriate, uncertain, and inappropriate scenarios.

For the appropriateness criteria, we created our own explicit scale for measuring the severity of COPD exacerbations because we did not find any in the literature. We based this scale on predictive factors for poor prognosis for COPD, recommendations from guidelines, and clinical knowledge of members of our research team [11,12].

### Development of the cohort study

A prospective cohort study was performed to validate the explicit criteria developed by the RAM. Other goals for the cohort study were to evaluate variability among hospitals in the appropriateness of hospital admission of patients experiencing COPD exacerbations and to study variability in access to care and outcomes. Sixteen hospitals belonging to the Spanish National Health Service agreed to participate: Hospital Costa del Sol, Hospital Valme, Hospital de Motril, Corporació Sanitaria Parc Taulí, Hospital del Mar, Hospital Universitario de La Princesa, Hospital Universitario Gregorio Marañón, Hospital Universitario La Paz, Hospital de Móstoles, Hospital Marqués de Valdecilla, Hospital Santa Marina, Hospital San Eloy, Hospital Galdakao-Usansolo, Hospital Txagorritxu, Complejo Hospitalario Donostia, and Hospital Cruces.

Patients attending the EDs of any of the 16 hospitals with an exacerbation of COPD were informed of the goals of the study and invited to voluntarily participate in it. All information was kept confidential. The Institutional Review Boards of the participating hospitals approved this project. Recruitment started in June 2008 and ended in September 2010.

Patients were candidates for the study if they presented to the ED of any of the participating hospitals with symptoms consistent of an exacerbation of COPD. Exacerbation was defined as an event in the natural course of the disease characterized by a change in the patient's baseline dyspnea, cough, and/or sputum that was beyond normal day-to-day variations, was acute in onset, and may have warranted a change in regular medication in a patient with underlying COPD [6]. Two possible presentations were considered:

#### Existing COPD

Patients were considered to have been previously diagnosed with COPD if they had a FEV_1_/forced vital capacity (FVC) quotient <70%, and a negative bronchodilation test with FEV_1 _change <200 mL and under 15% of the baseline value.

#### New COPD

Patients not previously diagnosed with COPD but in whom the disease was suspected were also eligible for inclusion in the study. This included smokers or former smokers of more than 15 packs per year with dyspnea, cough, or expectoration for more than three months per year, and experiencing symptoms resembling a clinical manifestation compatible with COPD exacerbation. The diagnosis had to be confirmed by spirometry within 60 days after the index episode at a time when the patient was stable, i.e., the absence of any increase in symptoms or changes in background therapy [13]. If a diagnosis of COPD was not confirmed, the patient was excluded from the study [6].

Patients were excluded from the study if they had COPD complicated by a comorbidity such as pneumonia, pneumothorax, or pulmonary embolism; lung cancer; or left cardiac insufficiency. Other exclusion criteria included a diagnosis of asthma, extensive bronchiectasis, sequelae of tuberculosis, pleural thickening, or restrictive diseases. Patients who did not wish to participate were also excluded.

### Data collected for the cohort study

A substantial amount of clinical and other data were needed to meet the objectives of the IRYSS-CAS. Data from several time points were needed: during the patient's evaluation in the ED; at the time the decision was made to hospitalize the patient or discharge him or her to home; in the medical ward (if needed); and during post-hospitalization or post-discharge follow-up. It must be noted that ED physicians were not asked to gather any information other than what they would usually collect for a patient experiencing an exacerbation of COPD. Instead, trained data managers gathered data from hospital and primary care medical records using a manual of instructions that aimed to standardize data collection.

Variables needed at each time point are listed in Table 1. Some of the information required a review of the patient's medical records. Patients admitted to the hospital were interviewed at 1 and 7 days after admission. Patients discharged from the ED to home were interviewed by telephone at, around, 1 and 7 days after discharge. All patients were interviewed by telephone 60 days after the index event.

**Table 1:** Main data needed at various time points for the IRYSS-COPD Appropriateness Study

Data needed at various time points
**Evaluation in the ED**
Arterial blood gases parameters including PO2, PCO2 and pH.
O2 saturation
Presence of diabetes mellitus
Presence of cardiovascular disease
Expected adherence to treatment
Response to previous treatments
Severity of baseline COPD
Number of hospital admissions in the year preceding the exacerbation
Need for oxygen therapy at home
Hemodynamic stability
Level of consciousness, measured by the Glasgow Coma Scale [14]
Presence of dyspnea at rest
Exercise tolerance in the ED
Respiratory rate
Presence of paradoxical breathing or use of accessory respiratory musculature
Sociodemographic information

**Decision to admit or discharge home**
Arterial blood gases parameters including PO2, PCO2 and pH.
O2 saturation
Hemodynamic stability
Level of consciousness, measured by the Glasgow Coma Scale[14]
Presence of dyspnea at rest
Respiratory rate
Presence of paradoxical breathing or use of accessory respiratory musculature

**If admitted, in the hospital**
Biological parameters such as arterial blood gases and blood glucose.
Respiratory rate
Presence of cardiac failure
Results of chest x-ray
Charlson Comorbidity Index[15]
Medications prescribed for the acute episode and subsequent admission, methods of administration, and number of days of intravenous drug therapy.
In-hospital morbidity and mortality: the appearance of complications, including all signs, symptoms, syndromes or diseases, that appeared or worsened during the hospital stay that were attributable to COPD or it treatment.
Death
Admission to an Intensive Care Unit (UCI) or to an Intermediate Respiratory Care Unit (IRCU); or need for Invasive Mechanical Ventilation (IMV) or Non-Invasive Mechanical Ventilation (NIMV)
Length of stay
General health status, from response to question 1 of the Short Form 36 (SF-36) questionnaire[16], degree of dyspnea, based on the Medical Research Council Dyspnea Index[17], and physical activity level (based on a scale employed previously in various studies at 1, and 7 days [18].
Quality of life, measured by the EuroQol-5D questionnaire at 1, and 7 days [19,20].

**Follow-up on admitted patients (after hospital discharge and up to 60 days post-discharge)**
General health status, from response to question 1 of the Short Form 36 (SF-36) questionnaire[16], degree of dyspnea, based on the Medical Research Council Dyspnea Index[17], and physical activity level (based on a scale employed previously in various studies) [18].
Quality of life, measured by the EuroQol-5D questionnaire [19,20].
Readmission within 30 days of the index exacerbation for the same reason, or readmission for any reason within 60 days after the index exacerbation
Complications, including all signs, symptoms, syndromes or diseases that appeared or worsened during the 60-day observation period attributable to COPD or its treatment
Variables collected from medical records in all patients with known COPD
Baseline severity of COPD as measured by FEV1
Hospital admissions during the previous 12 months
Baseline therapy (inhaled long-acting beta agonist, long-acting anticholinergics, inhaled corticosteroid and/or supplemental oxygen)
Presence of associated diseases such as diabetes, hypertension, ischemic heart disease and/or valve disease, cor pulmonale, peptic ulcer disease, psychiatric disorders, rheumatic disease, history of stroke or deep vein thrombosis, and others needed to determine the Charlson Comorbidity Index
Social support and level of functional dependency

**Following discharge home from the ED (1, 7, and 60 days post-discharge)**
General health status, from response to question 1 of the Short Form 36 (SF-36) questionnaire[16], degree of dyspnea, based on the Medical Research Council Dyspnea Index[17], and physical activity level (based on a scale employed previously in various studies at 1, 7, and at 60 days [18].
Quality of life, measured by the EuroQol-5D questionnaire at 1, 7, and at 60 days [19,20].
Charlson Comorbidity Index[15]
Medication use
Response to medications
Need for supplemental oxygen
Visits to the patient's primary care physician, subsequent ED visits or hospital readmissions
Death, complications, presence of other symptoms
Level of social support
Level of functional dependency

#### In the ED

As is true for almost any encounter in the ED, substantial information is gathered for a patient experiencing an exacerbation of COPD. The main data collected were those related to the patient's respiratory function (Arterial blood gases, respiratory rate, dyspnea), consciousness level measured by the Glasgow Coma scale, [14] background, and presence of other pathologies as those recorded in the Charlson Comorbidity Index [15]. Variables needed from the initial evaluation in the ED are listed in Table 1.

#### At the time of decision making

Data collected at the ED decision time was related to the patient's respiratory status at that moment as well as variables needed to create the appropriateness scenarios, determine the severity of the exacerbation, and evaluate other study criteria (Table 1).

#### In the hospital

For patients admitted to the hospital, we collected data directly from the patient's medical record and from a direct interview with him or her from the first day after admission until discharge (Table 1). Patients were interviewed about their general health status (response to question 1 of the Short Form 36 (SF-36) questionnaire[16]), degree of dyspnea, based on the Medical Research Council Dyspnea Index[17], physical activity level (based on a scale employed previously in various studies[18]) and also completed the EuroQol-5D [19,20]. Patients were also asked about social support and level of functional dependency. This information was recorded in the first 24 hours after arrival to the ED and at discharge.

#### Following discharge home from the ED

Among patients discharged home from the ED, telephone interviews were conducted around 1 day, 7 days, and 60 days after discharge to assess the level of dyspnea, physical activity, and general health (see previous description), the use of and response to medications, need for supplemental oxygen, the need for visits to the patient's primary care physician, subsequent ED visits or hospital readmissions, vital status, presence of other symptoms, social support, and level of functional dependency.

#### During follow-up

Data collected during follow-up included general health status (SF-36 question), degree of dyspnea, physical activity level, and quality of life, all as previously described. Readmission within 30 days of the index exacerbation for the same reason, or readmission for any reason between 31 and 60 days after the index exacerbation was recorded, as were complications, including all signs, symptoms, syndromes or diseases, which appeared or worsened during the 60-day observation period attributable to COPD or its treatment. For all patients with known COPD, additional variables collected from medical records include baseline severity of COPD as measured by FEV1; hospital admissions during the previous 12 months; baseline therapy (inhaled long-acting beta agonist, long-acting anticholinergics, inhaled corticosteroid and/or supplemental oxygen), the presence of associated diseases such as diabetes, hypertension, ischemic heart disease and/or valve disease, cor pulmonale, peptic ulcer disease, psychiatric disorders, rheumatic disease, history of stroke or deep vein thrombosis, and others needed to determine the Charlson Comorbidity Index[15].

### Definitions of outcome measures

The following outcomes were defined for the study during admission, as well as at 7 days, and 60 days after the index ED visit:

- Mortality.

- Very serious evolution of COPD, defined as any of the following added to mortality: ICU admission, the need for invasive mechanical ventilation, and/or cardiac arrest.

- Serious evolution of COPD, defined as any of the following added to very serious evolution: non-invasive mechanical ventilation for more than 2 days when mechanical ventilation was not needed before admission, and/or admission to an intermediate respiratory care unit for 2 or more days.

Several secondary outcomes were also defined:

- A variable called "complications" in which we noted the presence of shock, cardiac arrhythmia, myocardial ischemia, pulmonary embolism, pneumonia, pneumothorax, or decompensated diabetes [21].

- A variable called "complicated evolution," which included patients having serious evolution of a COPD exacerbation and/or complications described above.

-Medication use, which included medications needed during admission, including intravenous corticosteroids, diuretics, antibiotics, and oral medications.

-Length of hospital stay, defined as the difference between the day of discharge and the day of admission.

### Assumptions on missing information

In the IRYSS-CAS, participating ED physicians were not expected or asked to gather extra information beyond what they would ordinarily collect in their clinical practice, nor were they asked to use or complete special study forms. Thus, information for some patients was not recorded upon their arrival in the ED or at the time the decision was made to hospitalize or discharge the patient.

Most of the missing data from the decision-making interval were not random but were instead the result of routine clinical practice. For example, if an ED physician believes that a patient's clinical situation is normal based on arterial blood gases performed upon arrival in the ED, there is no need to repeat arterial blood gases at the time the decision is made to hospitalize or discharge the patient. In such cases, pulse oximetry was generally performed when the admission decision was made, or a second arterial blood gases was performed in the ward. Missing data regarding sociodemographic data, information regarding the patient's previous health situation, or variables essential for completing the appropriateness algorithm (Table 2) were considered to be random. Table 3 shows missing data for the clinical variables at arrival to the ED and at the time of decision making for admission or discharge.

**Table 2:** Available sociodemographic and clinical data collected at arrival to the ED (n = 2877)

	Number of patients	n (%)	% with missing data
Age*	2876	72.84 (9.51)	0.03
**Clinical data previous to arrival at the ED**			
Heart disease - Yes	2856	617 (21.6)	0.7
Diabetes - Yes	2858	619 (21.66)	0.7
Charlson Comorbidity Index	2846		1.1
0		68 (2.39)	
1		1116 (39.21)	
>1		1662 (58.40)	
Number of hospital admissions in the previous 12 months for COPD	2839		1.3
3 or more		360 (12.68)	
Less than 3		2479 (87.32)	
Basal FEV_1_%	2430		15.5
>= 50		809 (33.29)	
< 50		1621 (66.71)	
Use of oxygen at home - Yes	2841	984 (34.64)	1.3
**At arrival at the ED**			
Expected good adherence to treatment	2845	2681 (94.24)	1.1
Positive response to previous treatment - Yes	2849	2684 (94.21)	1.0
Edema - Yes	2712	516 (19.03)	5.8
Dyspnea upon arrival in the ED - Yes	2739	1834 (66.96)	4.8
Glasgow Coma Scale - altered	2874	77 (2.68)	0.1

*Represented as mean (std)

**Table 3:** Respiratory rate and arterial blood gases data available at arrival in the ED and the time a decision was made to admit or discharge to home.

	Without assumptions on missing data						With assumptions on missing data					
	**Arrival at ED**			**Decision time**			**Arrival at ED**			**Decision time**		

	**N**	**% missing**	**n(%)**	**N**	**% missing**	**n(%)**	**N**	**% missing**	**n(%)**	**N**	**% missing**	**n(%)**

Respiratory Rate	2314	19.6		1536	46.6		2621	8.9		2200	23.5	
<20			435 (18.80)			453 (29.49)			435 (16.60)			673 (30.59)
20-24			832 (35.96)			713 (46.42)			1139 (43.46)			1084 (49.27)
>24			1047 (45.25)			370 (24.09)			1047 (39.95)			443 (20.14)
pH	2494	13.3		747	74.0		2661	7.5		2410	16.2	
>=7.35			2141 (85.85)			626 (83.80)			2308 (86.73)			2227 (92.41)
7.26-7.35			293 (11.75)			105 (14.06)			293 (11.01)			160 (6.64)
<7.26			60 (2.41)			16 (2.14)			60 (2.25)			23 (0.95)
PO2	2469	14.2		742	74.2		2642	8.17		2228	22.6	
>60			1130 (45.77)			346 (46.63)			1303 (49.32)			1434 (64.36)
45 - 60			979 (39.65)			316 (42.59)			979 (37.06)			668 (29.98)
<=45			360 (14.58)			80 (10.78)			360 (13.63)			126 (5.66)
PCO2	2485	13.6		744	74.1		2485	13.6		1992	30.76	
<=45			1365 (54.93)			313 (42.07)			1365 (54.93)			1106 (55.52)
45-55			592 (23.82)			204 (27.42)			592 (23.82)			544 (27.31)
55-65			288 (11.59)			118 (15.86)			288 (11.59)			184 (9.24)
>65			240 (9.66)			109 (14.65)			240 (9.66)			158 (7.93)

To impute missing data when possible, we made the following assumptions:

#### Assumptions to recover missing data from ED arrival

There were very few missing arterial blood gases data from arrival in the ED, reducing assumptions to a minimum. In most cases, when arterial blood gases data were not available, O2 saturation data from pulse oximetry was available. It was used to impute missing arterial blood gases parameters as follows:

##### pH

If a patient's O2 saturation on arrival in the ED was higher than 94% without the use of supplemental O2 , then we assumed the pH to be >=7.35.

##### PO2

If a patient's O2 saturation on arrival in the ED was higher than 94% without the use of supplemental O2, PO2 was assumed to be >60 mmHg.

##### PCO2

No assumptions were made.

##### Respiratory frequency

If the patient's respiratory frequency was described in the medical record as eupneic, a respiratory frequency <20 breaths per minute was assumed. For patients described as tachypneic, those with paradoxical breathing, or those using accessory musculature to breathe, a respiratory frequency >=20 was assumed.

#### Assumptions to recover missing data at the time of decision making

We assumed that most of the missing data from the time the decision was made to admit or discharge the patient were not random but were generally due to physicians' prior knowledge of data collected when the patient arrived or their clinical evolution in the ED. For example, 79% of the missing data on pH at the time of decision making belonged to patients whose pH was >=7.35 when they arrived in the ED, and only 21.5% of patients whose pH was >=7.35 when they arrived in the ED had a second arterial blood gases done. Patients with a very severe exacerbation of COPD had repeated arterial blood gases performed in the medical ward or ICU. We recovered some missing information from other available data by making the following assumptions, which in the analysis are planned to be done sequentially as described below:

##### pH

If the patient arrived in the ED with a pH >=7.35, and the PO2 was >60 mmHg or the O2 saturation was >=90%, and the O2 saturation was >94% when the decision was made to admit or discharge, and the patient was discharged home, the pH at the decision time point was assumed to be normal (>=7.35). If the patient arrived in the ED with a pH >=7.35, and the PO2 was >60 mmHg or the O2 saturation was >=90%, and the O2 saturation was >94% when the decision was made to admit or discharge the pH at the decision time point was assumed to be normal (>=7.35). If the patient arrived at ED with a PH>=7.35 and was admitted and the O2 saturation at the arrival at the medical ward was >94 then we assumed the PH at the decision making time to be >=7.35. Finally, for patients who were admitted to the hospital, we used the pH recorded at the arrival at the medical ward.

##### PO2

To impute missing PO2 at the time of decision making, we first turned to O2 saturation data from pulse oximetry recorded near the decision point. If O2 saturation was >94%, we assumed that the PO2 was >60 mmHg; if O2 saturation was <90%, the PO2 was assumed to be >45 and <=60 mmHg. If the patient was discharged home and the PO2 at arrival was >60 then PO2 was assumed at the time of decision making to be >60. If O2 saturation was not available near the decision point and the patient was admitted, PO2 recorded at the arrival at the medical ward was used as the value at decision making. If PO2 was not recorded on admission to the medical ward, but O2 saturation was available and this was >94%, then we assumed that the PO2 was >60 mmHg; for O2 saturation <90%, the PO2 was considered to be >45 mmHg and <=60 mmHg.

##### PCO2

If the patient was discharged home and the PCO2 at arrival was <=45 then we assumed the PCO2 at decision making to be <=45. If the patient was admitted, we used the PCO2 recorded at the arrival at the medical ward when available.

##### Respiratory rate

As was the case for ED arrival, if the patient's exact respiratory frequency was not recorded at the decision point, a respiratory frequency of <20 breaths per minute was assumed for patients described as eupneic while a respiratory frequency >20 breaths per minute was assumed for those described as tachypneic, those with paradoxical breathing, or those using accessory musculature to breathe. If the patient was discharged home having a respiratory frequency at arrival normal (<20) we assumed a normal respiratory frequency to be at the decision time. If the patient was admitted and the respiratory rate was available at the arrival at the medical ward, we used this data as the value at the time of decision making.

### Sample size estimation

Sample size estimations were made for some of the main hypotheses. To detect a difference in the rate of inappropriate admissions between the centers with the best rates (<10%) and those with the worst rates (>20%), 155 patients would be needed for α = 0.05 and1-ß = 0.80. To develop predictive models for serious or complicated evolution of COPD, we needed at least 10 instances of the dependent variable of interest for each independent variable included in the multivariate logistic regression [22]. With 10 independent variables in the multivariate logistic regression model, at least 100 such events of the dependent variable in the sample from which we derived the prediction rule would be needed to ensure that the logistic regression model converges properly. Previous data from some of our participating centers indicated that the number of events of our dependent variable would be not more than 20% of patients admitted with COPD exacerbations. Therefore, to test this hypothesis, we needed to recruit at least 1,000 patients to have enough events in half the total sample which could constitute the derivation sample.

In summary, we planned to recruit at least 2,500 patients to answer the study's hypotheses.

### Statistical Analysis

We used means and standard deviations (SDs), frequencies, and percentages to describe the sample. Sociodemographic, clinical data before attending the ED and at the ED as well as main outcomes were compared between patients with missing data on arterial blood gases variables (on the pH) at the ED decision making time, versus those with complete data, by using the Chi-square or Fisher's exact tests for categorical variables and the t-test or the Wilcoxon nonparametric test when necessary.

To make clinical interpretation easier, we first categorized variables based on commonly used clinical criteria [6,13,23] and clinical expertise [13], and confirmed them by the following statistical techniques[24].

- PCO2: <=45, 46 to 55, 56 to 65 and >65 mmHg

- pH: <=7.25, 7.26 to 7.34, and >=7.35

- PO2: >60, 46 to 60, and <=45 mmHg

- O2 saturation measured by pulse oximetry: >=94% or <90%

- Respiratory rate: >15 to <20; 20 to 24; and >=25 breaths per minute

We then developed models of variables influencing very serious evolution of COPD and serious evolution using generalized additive models (GAM), searching for cut-point estimation with smooth functions. When necessary, a confidence interval was calculated for the cut-point. The statistical techniques we used to explore the relationship between covariate and outcome have been covered in two steps:

1. We used GAM to graphically display the relationship between each predictor and evolution of COPD, the selected outcome of interest [25]. Each predictor was included in a GAM in a flexible way, using P-splines as smoothers [26]. These graphical displays were used to select adequate cut-points of the continuous predictors in order to make them categorical predictors. Cut-points were selected based on the curvature and the slope of the smooth curve that estimates the relationship of each predictor with the outcome, considering the points where the smooth curve crosses the x axis.

2. If the relationship between the predictor and the outcome was linear, as is the case for respiratory frequency, cut-points were selected based on the value where the line crosses the x axis. This value was selected as the zero risk point and a 95% prediction confidence interval was built around it using a regression logistic model. This yielded three categories: a middle one indicating no risk flanked by a lower one indicating lower risk for the outcome and an upper one indicating higher risk.

For variables where assumptions were made (arterial blood gases parameters-pH, PCO2 and PO2- and respiratory rate) at ED arrival or at ED decision time to discharge home or admit the patient to the hospital, we checked all the previously described assumptions (in the same order as described) in the sample of patients who had complete data in all points in time of the study.

All variables defined as outcomes of the study were categorized as dichotomous: presence or absence.

### Description and Characteristics of the IRYSS-COPD Appropriateness Study (IRYSS-CAS) cohort

A total of 3,276 patients were recruited for the study. Of these, 198 (6%) were excluded because their COPD was complicated by other major pathologies at the time of ED admission. Fifty-six patients of the patients who entered the study as new diagnosis of COPD were excluded when the diagnosis was not confirmed by spirometry within 60 days of the index episode. Another 145 patients (4.4%) were lost during the follow-up period.

**Figure 1:**
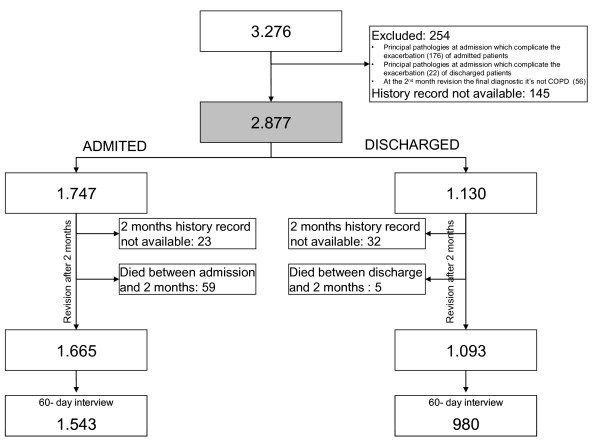
**Exclusions and losses during all the study**.

Thus, the final study population included 2,877 patients who attended one of the 16 participating EDs with an exacerbation of COPD. Of these, 1,747 patients (60.7%) were admitted to the hospital and 1,130 (39.3%) were discharged home from the ED (see Figure 1). At follow up at 2 months after the ED index visit, 88.3% of patients admitted to the hospital completed the interview and had their medical record available, compared with 86.7% of those discharged home.

Sociodemographic and clinical variables from the arrival in the ED time were mostly complete (Table 2). Two key variables in the clinical decision making process for COPD-- arterial blood gases and respiratory rate--were available for more than 80% of the patients arrival at the ED. After applying assumptions about missing information, these data were missing for only 5% to 10% of patients. Missing data were much more common at the time the decision was made to admit or discharge (e.g., 74% missing data for arterial blood gases). After applying assumptions, that was reduced to 16% to 31% (Table 3). For the main outcomes of our study, data were complete, with less than 1% of missing data in most cases. No assumptions were made for these missing variables (Table 4). For cases where assumptions were made (arterial blood gases and respiratory rate) at ED arrival or at ED decision time to discharge home or admit the patient to the hospital we checked all the previously described assumptions (in the same order as described) in the sample of patients who had complete data in all points in time of the study. Results of this validation process are included in Table 5. We, then, study the differences between those patients having arterial blood gases information (pH) at the time the decision to admit to the hospital or discharge home, with those patients who did not have such information available (Table 6). Patients with arterial blood gases information at the decision time had poorer health status (on basal FEV1%, previous admissions, use of oxygen at home; and dyspnea, Glasgow score and arterial blood gases at arrival) and outcomes (admission to an intensive care unit (ICU), admission to an intermediate respiratory care unit (IRCU), need for invasive mechanical ventilation (IMV) , non-invasive mechanical ventilation (NIMV) or those with chronic oxygen therapy in the medical ward) than those with missing data on those parameter.

**Table 4:** Data available on outcome variables

	Outcomes			
	**Without Assumptions on Missing Data**		**With Assumptions on Missing Data**	

	**N**	**% miss**	**N**	**Outcome present** **n(%)**

Death	2852	0.87%	2877	60 (2.09)
Admission to Intensive Care Unit	2858	0.66%	2877	27 (0.94)
Invasive Mechanical Ventilation	2856	0.73%	2877	38 (1.32)
Admission to intermediate respiratory care unit	2858	0.66%	2877	74 (2.57)
Noninvasive mechanical ventilation	2840	1.29%	2877	100 (3.48)
Chronic oxygen therapy in the medical ward	2848	1.01%	2877	1512 (52.55)
Cardiac arrest	2789	3.06%	2877	20 (0.70)

**Table 5:** Validation of missing data assumptions on complete data patients

	Validation of the assumptions made at arrival	N and % of missing data recovered with each assumption	Validation of the assumptions made at the decision time	N and % of missing data recovered with each assumption
Respiratory Rate	1. 91%	1. 307 (54.5%)	1. 88% 2. 90% 3. 64% (when FR<20), 70% (when FR[20-24]) and 58% (FR>24)	1. 263 (19.6%) 2. 134 (10%) 3. 267 (19.9%)

pH	1. 90%	1. 167 (43.6%)	1. 97% 2. 98% 3. 98% 4. 91% (pH>=7.35), 63% (pH [7.26-7.35]) and 50% (pH<7.26)	1. 937 (44%) 2. 146 (6.9%) 3. 181 (8.5%) 4. 399 (18.7%)

PO2	1. 95%	1. 173 (42.4%)	1. 90% for O2 saturation >94%; 70% for O2 saturation <90% 2. 91% 3. 77% (PO2>60), 82% (PO2 (45-60]) and 63% (PO2 <=45) 4. 63% for O2 saturation >94%; 64% for O2 saturation <90%	1. 637 (29.8%) 2. 342 (16%) 3. 386 (18.1%) 4. 121 (5.7%)

PCO2	No assumptions were made		1. 90% if PCO2 at arrival normal 2. 86% (PCO2 <=45), 73% (PCO2 (45-55]) , 75% (PCO2 (55-65]) and 80% (PCO2>65)	1. 714 (33.5%) 2. 534 (25%)

Numbers in cells indicated, first, the order of the assumption, as included in the manuscript text, and, then, the percentage of patients with complete data that fulfill that assumption.

**Table 6:** Comparison of sociodemographic, clinical and outcome parameters among patients with arterial blood gases data available at decision making versus those with missing data at that time.

	Patients without missing data N = 747	Patients with missing data N = 2130	p value
Age*	72.54 (9.50)	72.94 (9.51)	0.3311
**Clinical data previous to arrival at the ED**			
Charlson Index			0.5522
<=1	301 (40.68)	883 (41.93)	
>1	439 (59.32)	1223 (58.07)	
Number of hospital admissions in the previous 12 months for COPD			<.0001
3 or more	128 (17.34)	232 (11.04)	
Less than 3	610 (82.66)	1869 (88.96)	
Basal FEV_1_%			<.0001
>= 50	169 (25.61)	640 (36.16)	
< 50	491 (74.39)	1130 (63.84)	
Use of oxygen at home - Yes	336 (45.41)	648 (30.84)	<.0001
**At arrival at the ED**			
Glasgow Coma Scale - altered	34 (4.55)	43 (2.02)	0.0002
Edema - Yes	153 (21.37)	363 (18.19)	0.0627
Dyspnea upon arrival in the ED - Yes	546 (76.36)	1288 (63.64)	<.0001
PH at arrival			<.0001
>=7.35	462 (69.79)	1679 (91.65)	
7.26-7.35	156 (23.56)	137 (7.48)	
<7.26	44 (6.65)	16 (0.87)	
PO2 at arrival			<.0001
>60	229 (35.12)	901 (49.59)	
45 - 60	273 (41.87)	706 (38.86)	
<=45	150 (23.01)	210 (11.56)	
Respiratory rate at arrival			<.0001
<20	79 (12.38)	356 (21.24)	
20-24	209 (32.76)	623 (37.17)	
>24	350 (54.86)	697 (41.59)	
PCO2 at arrival			<.0001
<=45	243 (36.82)	1122 (61.48)	
46-55	165 (25.00)	427 (23.40)	
56-65	117 (17.73)	171 (9.37)	
>65	135 (20.45)	105 (5.75)	
**Outcome parameters**			
Death	18 (2.41)	42 (1.97)	0.4712
Admission to Intensive Care Unit	14 (1.87)	13 (0.61)	0.0021
Invasive Mechanical Ventilation	16 (2.14)	22 (1.03)	0.0223
Admission to Intermediate respiratory care unit	50 (6.69)	24 (1.13)	<.0001
Noninvasive mechanical ventilation	57 (7.63)	43 (2.02)	<.0001
Chronic oxygen therapy in the medical ward	476 (63.72)	1036 (48.64)	<.0001
Cardiac arrest	6 (0.80)	14 (0.66)	0.6796

*Mean (Std)

Patients were included in the patients with missing data at decision making time group if they did not have the pH data available at that time. If they had it, they were included in the group with no missing data at that time.

**Figure 2:**
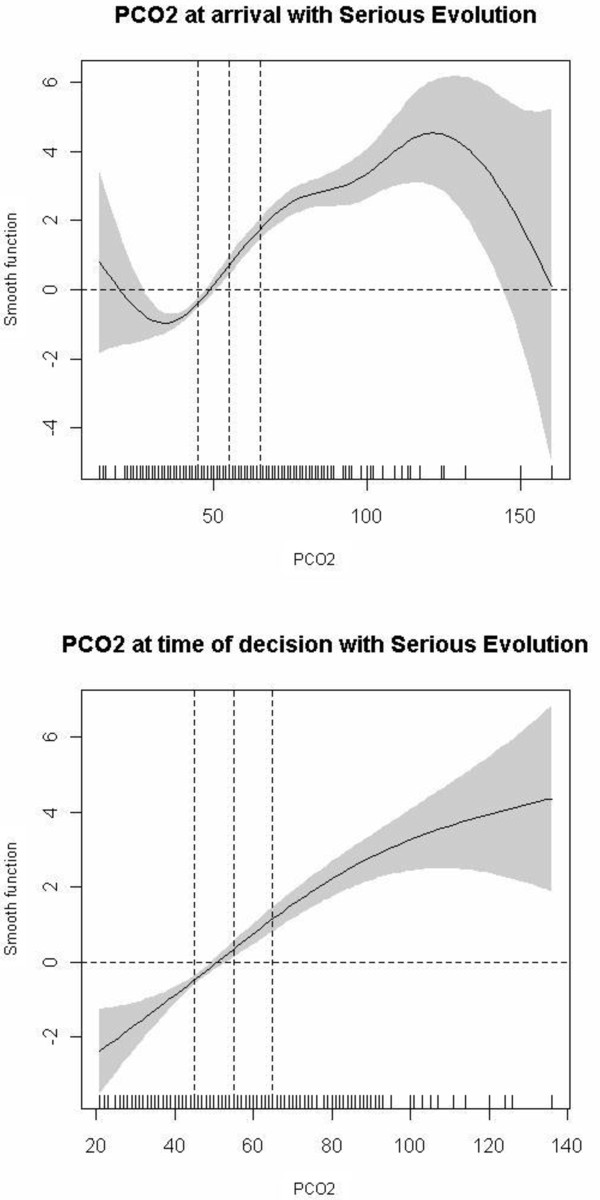
**Relationship of PCO2 at ED arrival and the decision to admit or discharge with serious evolution of COPD in the short follow-up**.

**Figure 3:**
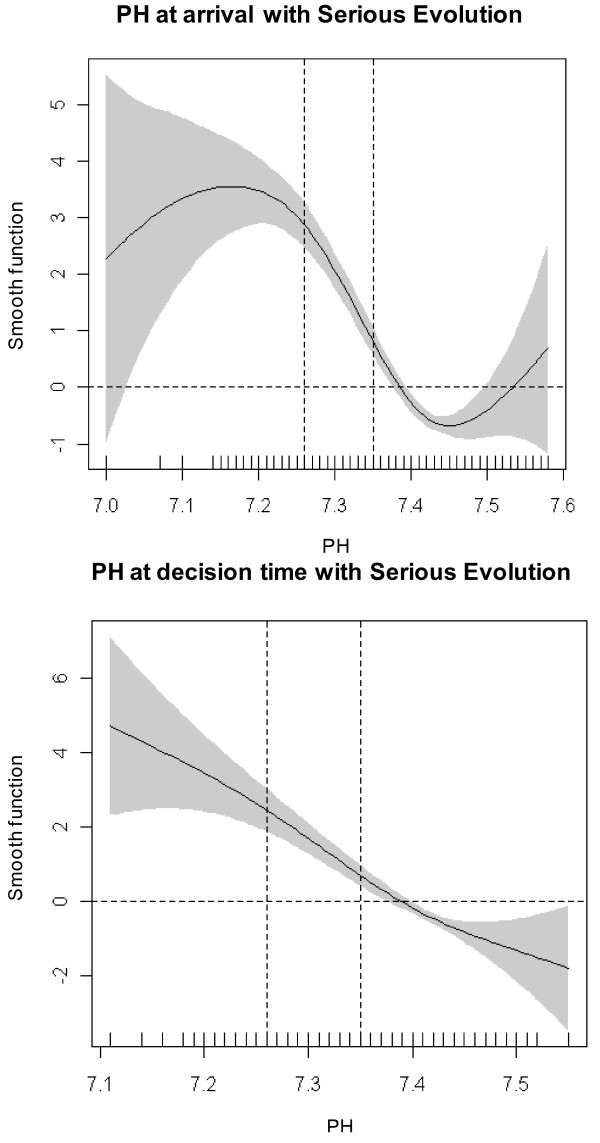
**Relationship of pH at ED arrival and the decision to admit or discharge with serious evolution of COPD in the short follow-up**.

**Figure 4:**
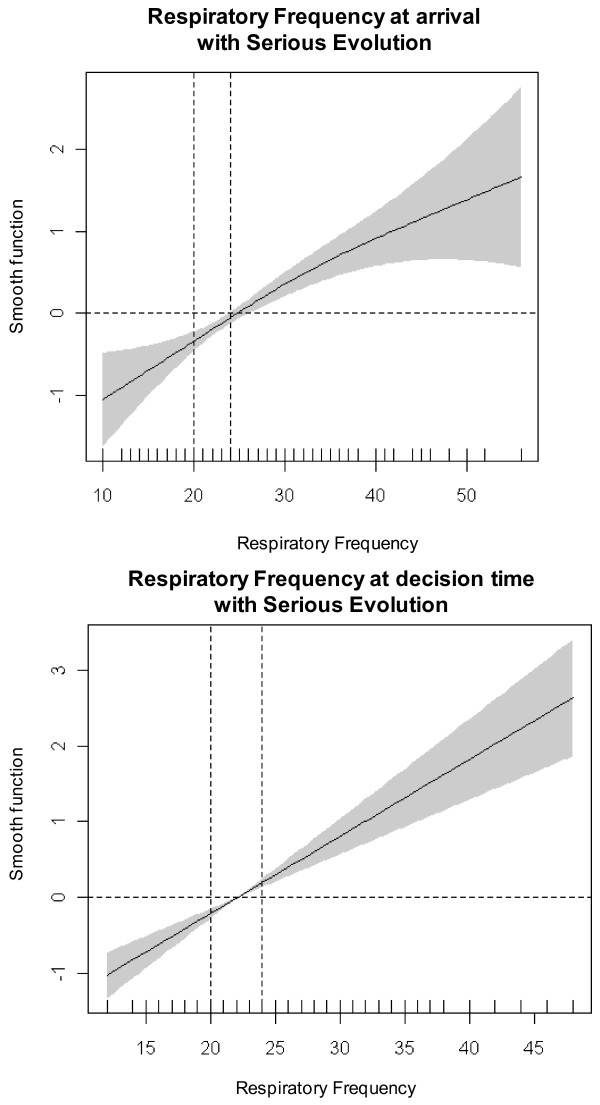
**Relationship of respiratory rate ED arrival and the decision to admit or discharge with serious evolution of COPD in the short follow-up**.

We also descriptively explored the relationship between the main arterial blood gases parameters (PCO2, pH) and respiratory rate at the time of arrival in the ED and at the time the decision was made to admit or discharge with the variable "serious evolution of COPD" as well as the relationship of their respective cut-off points (Figures 2, 3, 4) in order to confirm visually if the commonly used cut-points for these parameters were associated with serious evolution of COPD over short-term follow-up (for patients discharged home from the ED, up to 7 days after discharge; for patients admitted to the hospital, until discharge from the hospital in admitted patients). As can be seen in the figures, the data confirm the use of these commonly used cut-points to established normality or increased risk of serious evolution of COPD.

## Discussion

Little evidence-based guidance is available to ED physicians when deciding whether to hospitalize a patient experiencing an exacerbation of COPD or to discharge him or her to home from the ED. In the absence of clinical trial data, other methods are needed to establish evidence-based processes of care for this common clinical scenario. We approached this problem by creating a set of appropriateness criteria for hospitalization using the RAND/UCLA Appropriateness Methodology and then applying these criteria in a prospective study conducted in the EDs of 16 hospitals.

Data collection in the prospective study posed some unique problems. In order to complete the appropriateness criteria algorithm, data were needed from the ED at two points in time--patient arrival in the ED and when the decision was made to admit the patient to the hospital or to discharge him or her to home from the ED. This presented a challenge because of missing data at one or both of these time points. For example, arterial blood gases information was missing for 74% of patients at the time the decision was made to admit or discharge home. This is largely due to physician knowledge of data collected when the patient arrived in the ED--if a patient's arterial blood gases were normal upon arrival in the ED, there is generally no need to carry out a second arterial blood gases at the time the decision is made to hospitalize or discharge the patient. We made several pre-defined assumptions that allowed us to reliably impute missing data in many instances.

To the best of our knowledge, no prior studies have focused on the development or validation of admission criteria for patients experiencing exacerbations of COPD. Although Roche et al. developed predictive models to create severity scores for such patients,[9] their score does not include arterial blood gases and other relevant data from the ED, which limits their results.

The IRYSS-CAS has several strengths. One is the use of the systematic, validated RAND/UCLA Appropriateness Methodology for creating appropriateness criteria for the hospitalization of patients experiencing COPD exacerbations. Another strength is our effort to validate these criteria in a large prospective cohort of patients recruited from 16 different EDs.

Limitations of the study must also be noted. As in any prospective cohort study, patients lost to follow-up is an issue. Among patients admitted to the hospital, 11.7% did not complete the 60-day interview, compared with 13.3% of those discharged home. This is similar to rates seen in other large multicenter clinical studies [27]. Missing data for some key variables is another limitation. In a study like the IRYSS-CAS, clinical practice prevails over research requirements. Thus, we had to work with the information routinely collected in the ED for patients experiencing COPD exacerbations. We created a set of predefined assumptions that allowed us to impute some missing data. These assumptions always considered the criteria used in clinical practice and took a conservative approach. It must also be noted that the patient population was almost entirely comprised of men (97%). Similar gender distributions have been observed in other studies performed in our country [28], which probably reflects our smoking patterns in the mid-20^th ^century. Although we do not consider this to be a serious limitation, it could affect the generalizability of the results.

In summary, the IRYSS-CAS will better delineate the requirements for hospital admission or discharge home for patients experiencing exacerbations of COPD and also provide a better understanding of the determinants of outcomes of COPD exacerbations. From a health service research perspective, the study will also help evaluate differences in appropriateness of hospitalization for COPD exacerbation between hospitals, as well as differences in outcomes between hospitals and their relationships with other parameters, such as sociodemographic or clinical variables.

## Competing interests

The authors declare that they have no competing interests.

## Authors' contributions

JMQ: participated in the conception and design, analysis and interpretation of the data, drafting of the article, critical revision of the article for important intellectual content, final approval of the article and obtaining of funding. CE: participated in the conception and design, analysis and interpretation of the data, drafting of the article, critical revision of the article for important intellectual content, and final approval of the article. SGG: participated in the conception and design, analysis and interpretation of the data, drafting of the article, critical revision of the article for important intellectual content, and final approval of the article. IB: participated in the analysis and interpretation of the data, statistical expertise, drafting of the article, critical revision of the article for important intellectual content, and final approval of the article. NG: participated in the design, provision of patients, collection and assembly of data, analysis and interpretation of the data, drafting of the article, critical revision of the article for important intellectual content, and final approval of the article logistic support. IA: participated in the analysis and interpretation of the data, statistical expertise, drafting of the article, critical revision of the article for important intellectual content, and final approval of the article. IL: participated in the design, provision of patients, collection and assembly of data, analysis and interpretation of the data, drafting of the article, critical revision of the article for important intellectual content, and final approval of the article logistic support

MB: participated in the design, provision of patients, collection and assembly of data, analysis and interpretation of the data, drafting of the article, critical revision of the article for important intellectual content, and final approval of the article logistic support and obtaining of funding. JAB: participated in the design, provision of patients, collection and assembly of data, analysis and interpretation of the data, drafting of the article, critical revision of the article for important intellectual content, and final approval of the article logistic support and obtaining of funding

SV: participated in the design, provision of patients, collection and assembly of data, analysis and interpretation of the data, drafting of the article, critical revision of the article for important intellectual content, and final approval of the article logistic support and obtaining of funding. The IRYSS- COPD Group participated in the design, provision of patients, collection and assembly of data, analysis and interpretation of the data, drafting of the article, critical revision of the article for important intellectual content, and final approval of the article logistic support. All authors read and approved the final manuscript.

## Pre-publication history

The pre-publication history for this paper can be accessed here:

http://www.biomedcentral.com/1472-6963/11/322/prepub
